# Supplementary material for: Assessment of Beeswax Adulteration by Paraffin and Stearic Acid Using ATR-IR Spectroscopy and Multivariate Statistics—An Analytical Method to Detect Fraud
Source: Foods. 2024 Jan 12;13(2):245. doi: 10.3390/foods13020245 (PMC10814946; doi:10.3390/foods13020245)
Supplement: Supplementary file 1 [file foods-13-00245-s001.zip › foods-2786161-supplementary.pdf]

# Assessment of Beeswax Adulteration by Paraffin and Stearic Acid Using ATR-IR Spectroscopy and Multivariate Statistics—An Analytical Method to Detect Fraud

**Konstantinos Chatzipanagis [1], Jone Omar [1,2] and Ana Boix Sanfeliu [1,*]**

[1] European Commission, Joint Research Centre (JRC), 2440 Geel, Belgium
[2] Analytical Development, Therapeutics Development & Supply, Discovery Product Development and Supply, Janssen Pharmaceutical Companies of Johnson and Johnson, 2340 Beerse, Belgium
* Correspondence: ana.boix-sanfeliu@ec.europa.eu

**Table S1.** Paraffin and stearic acid calibration samples.

| Paraffin | CP1 | CP2 | CP3 | CP4 | CP5 | CP6 | CP7 | CP8 |
|---|---|---|---|---|---|---|---|---|
| Paraffin concentrations (% w/w) | 0.00 | 5.02 | 9.69 | 15.10 | 20.07 | 25.15 | 49.69 | 100 |
| **Stearic acid** | **CA1** | **CA2** | **CA3** | **CA4** | **CA5** | **CA6** | **CA7** | **CA8** |
| Stearic acid concentrations (% w/w) | 0 | 0.68 | 1.14 | 4.9 | 14.89 | 25.65 | 50.64 | 100 |

Figure 5 indicates that samples 16 and 22 may contain amounts of tri-stearin and/or beef fat, in addition to paraffin and/or stearic acid. An independent way of investigating this assumption is illustrated in Figure S1, where the experimental IR spectrum of sample 22 and a calculated spectrum to simulate the former are presented. The calculated spectrum is computed by linear combinations of the reference spectra of beeswax, stearin, paraffin and stearic acid. Each reference spectrum was multiplied with a weighting factor and then all four weighted spectra were added together. The weighting factor is considered as a rough approximation of the presence of each substance. The linear combination used in Figure S1 is the following:

Simulated spectrum = (0.4*Beeswax) + (0.29*Paraffin) + (0.25*Stearin) + (0.06*Stearic Acid)

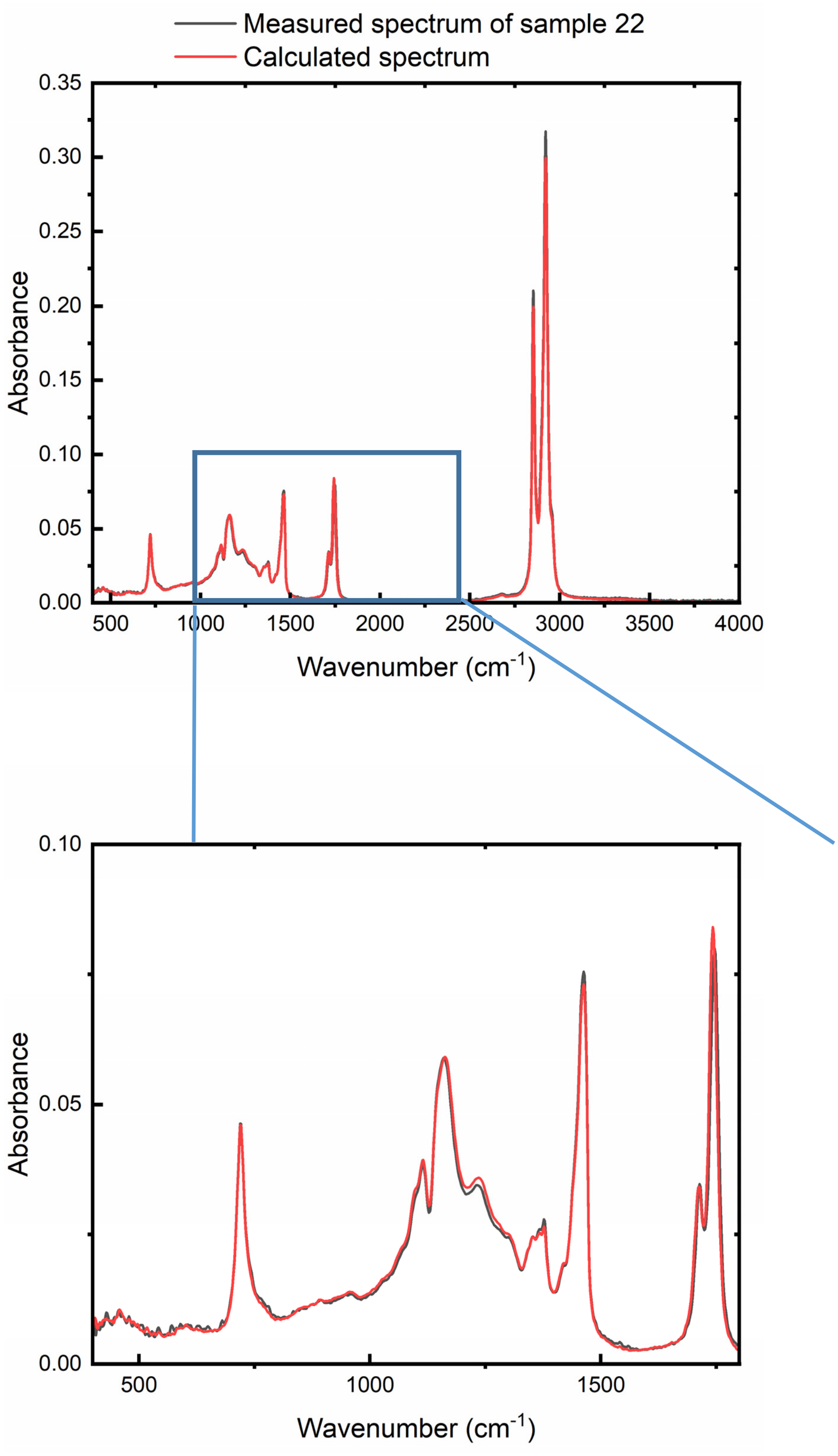


**Figure S1**. **Top:** Comparison of the experimental and simulated IR spectra in the 400 – 4000 cm$^{-1}$ range**. Bottom:** Enlarged view of the same spectra in the 400 – 1800 cm$^{-1}$ range for better visualization.
